# Efficient implementation of a local tomography reconstruction algorithm

**DOI:** 10.1186/s40679-017-0038-1

**Published:** 2017-01-19

**Authors:** Pierre Paleo, Alessandro Mirone

**Affiliations:** 1 ESRF-The European Synchrotron, 71 avenue des Martyrs, 38043 Grenoble, France; 2 Université de Grenoble, 11 Rue des Mathématiques, 38400 Saint-Martin-d’Hères, France

**Keywords:** Tomography, Interior problem, Local tomography, Reconstruction algorithm

## Abstract

We propose an efficient implementation of an interior tomography reconstruction method based on a known subregion. This method iteratively refines a reconstruction, aiming at reducing the local tomography artifacts. To cope with the ever increasing data volumes, this method is highly optimized on two aspects: firstly, the problem is reformulated to reduce the number of variables, and secondly, the operators involved in the optimization algorithms are efficiently implemented. Results show that $4096^2$ slices can be processed in tens of seconds, while being beyond the reach of equivalent exact local tomography method.

## Background

Computed tomography is a permanently evolving X-ray imaging technique finding various applications from medical imaging to materials science and non-destructive testing [1]. From a series of radiographs acquired at various angles, the interior of the scanned volume is reconstructed. In the ideal case, i.e., with a sufficient signal-to-noise ratio and a proper modeling, the reconstruction can be computed relatively easily. However, experimental constraints usually move away from the ideal case and require more advanced reconstruction methods. Among these constraints is the imaging of an object bigger than the detector field of view. This setup is called *local tomography* or region-of-interest (ROI) tomography.

**Fig. 1:**
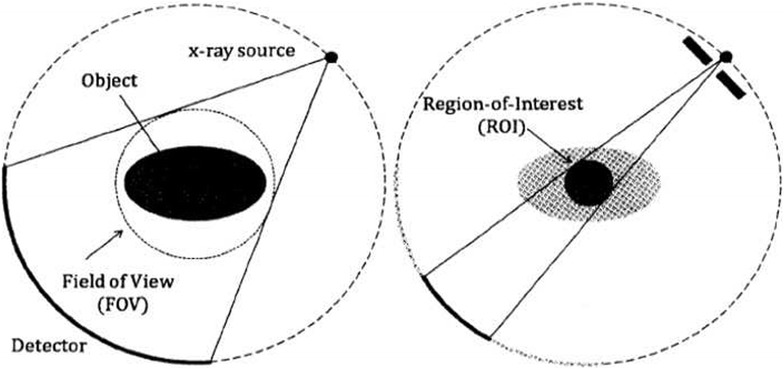
In a standard tomography setup (*left*), the detector field of view is large enough to image the whole object, as all the X-rays passing through the object hit the detector for all angles. In a local tomography setup (*right*), only X-rays passing through the region of interest hit the detector for all angles. Image from [12].

In local tomography, the detector measures rays coming out of the imaged ROI, and also contributions from the external part, as depicted in Fig. 1. As the external parts are not imaged for every angle, the data are incomplete. This incompleteness is the challenge of local tomography, for it can be shown that the object cannot be stably reconstructed from the acquired data, even in the ROI [2]. The problem of reconstructing a ROI embedded in a wider object is called the *interior problem*. The interior problem has infinitely many solutions in general, in the sense that a solution can differ from another solution by an infinitely differentiable function [3].

Local tomography methods basically consist in estimating the exterior of the ROI from the acquired measurements. This can be done with sinogram extrapolation (see for example [4, 5]) or in the slice domain. These methods, although they can yield satisfactory results, are only heuristics in general. Solutions computed with these methods often suffer from the *cupping effect*, which is an artifact appearing as a low-frequency bias.

Theoretical investigations, however, found that, with a prior knowledge on region of interest, the interior problem can be solved [6]. This prior knowledge can be about the values of a subregion of the ROI or about the nature of the solution [7, 8].

In this work, we consider a reconstruction method described in [9] where a prior knowledge is available as values of a subregion. We show how the reduction of the number of unknowns can be coupled with an efficient implementation of the involved operators, in order to cope with the scales of modern datasets.

## Methods

Based on the observation that the filtered backprojection with extrapolation provides satisfactory reconstruction of medium and high frequencies of the slice, the method aims at improving the reconstructed slice by removing the local tomography artifacts visible as low-frequency artifacts (cupping effect). This correction is performed by representing the reconstruction error in a coarse basis, reducing the number of degrees of freedom of the problem.

From an exact iterative reconstruction method, the reconstruction problem is reformulated to incorporate the local tomography setup, the prior knowledge constraint and the representation of the image in a coarse basis. Each operator of the forward model is analyzed to enable an efficient implementation. Notably, the projector is reduced to a point-projector which is efficiently implemented with a sparse matrix-vector multiplication.

The local reconstruction implementation is validated on simulated data for which the cupping effect is prominent. The proposed method is compared against another exact local reconstruction method also based on known region. Two criteria are compared: the number of required iterations to achieve an acceptable reconstruction, and the total execution time. The former reflects the relative ill-posedness of the problem and the performance of the chosen optimization algorithm, while the latter shows how the efficient implementation of the operators affects the reconstruction time. The benchmarks are carried on data compatible with modern data volumes, up to $4096^2$ pixels with 4000 projections.

## An iterative correction algorithm for local tomography

### Local tomography and artifacts

The most common local tomography reconstruction method is extrapolating the sinogram before computing the filtered backprojection (FBP), hereafter denoted *padded FBP*. The extrapolation is usually done by replicating the sinogram boundary values. This prevents truncation artifact (Gibbs phenomenon) from occurring, and often provides acceptable results [10].

However, this technique can fail when the ROI is surrounded by anisotropic and/or strongly absorbing material or when the reconstruction has intrinsically low contrast (for example different parts with the same linear absorption coefficient).

The notable local tomography artifact is the cupping effect. On a reconstructed image, local tomography artifacts appear as a varying contrast. The gray values are typically higher far from the center than close to the center, forming a “cup.” The cupping is also visible when plotting an image line passing through the center, as a function of the pixel location. Such lines are hereby called *profiles*, for example, the vertical line profile is the vertical line of the image passing through the center.

**Fig. 2:**
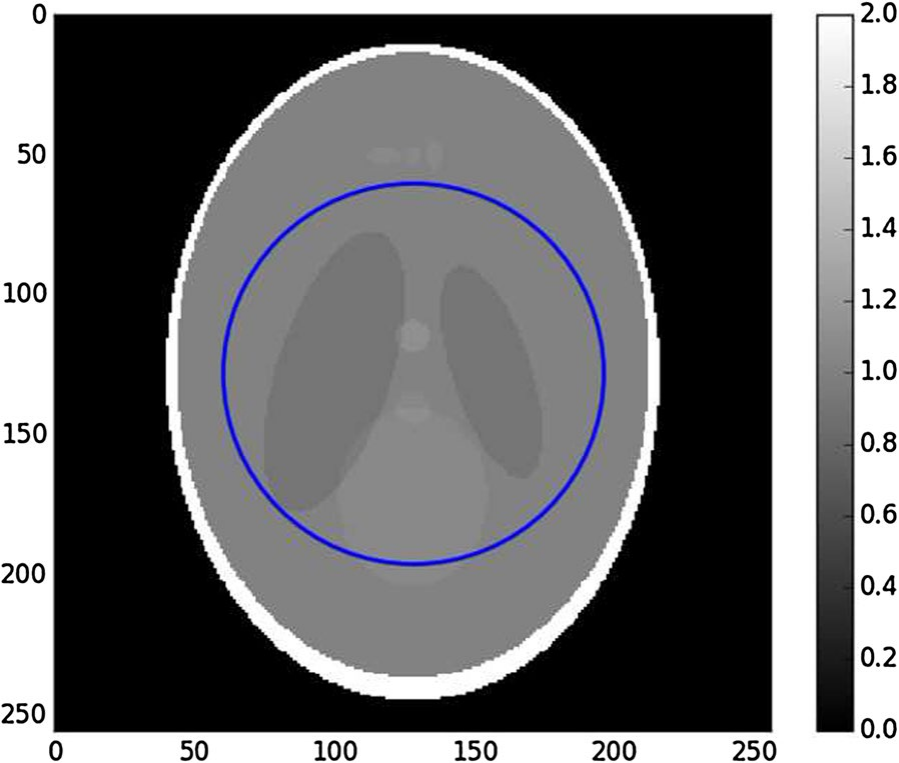
Shepp–Logan phantom, $256^2$ pixels. The *right bar* indicates the gray values, which for real data can be the linear attenuation coefficient values. The *blue circle* is the region of interest covered by the detector field of view

**Fig. 3:**
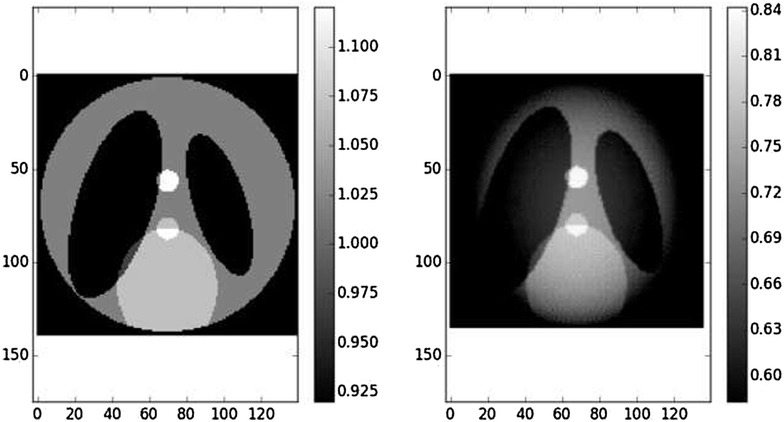
Zoom on the region of interest defined by the blue circle in Fig.  2. The support is $136^2$ pixels. *Left* ground-truth zoom. *Right* reconstruction with padded FBP. The image is brighter close to the center than far from the center, which is characteristic of the cupping effect. Contrast was adapted with respect to the center of the images

**Fig. 4:**
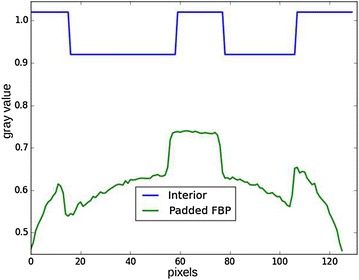
Horizontal line profile in the region of interest. *Blue* ground truth. *Green* padded FBP reconstruction. The error on the padded FBP reconstruction appears as a low-frequency mean bias

Figure 2 shows the Shepp–Logan phantom with a region of interest. Figure 3 shows the reconstruction with padded FBP, and Fig. 4 shows line profile of the reconstruction. The cupping effect is clearly visible in both the reconstruction image and profile. This cupping effect can be detrimental for the post-reconstruction analysis, for example, segmentation.

In this work, we examine a family of exact reconstruction methods based on a known subregion. We implement a method handling a reduced number of unknowns by expressing the image in a coarse basis in order to correct the cupping effect.

### Iterative reconstruction

Iterative methods in tomography are based on optimization algorithms solving problem (1)

1 $$\begin{aligned} P x = d \end{aligned}$$

where *P* is the model of the projection operator, *d* the acquired data, and *x* is the unknown volume to recover. In the remainder of this paper, we consider reconstruction of a single slice rather than a volume, so *x* shall denote two-dimensional slices. In parallel beam geometry, as it is the case in synchrotrons, reconstruction can be performed by reconstructing the slices independently.

In this context, the reconstructed slice *x* is an image of support $N \times N = N^2$, where *N* is the number of pixels of the detector horizontally. The sinogram *d* support is $N \times N_p$, where $N_p$ is the number of projections. Thus, the projector is theoretically an operator of dimensions $(N \times N_p, \, N^2)$, assuming that slices are stacked as one-dimensional $N^2$ vectors, and sinograms are stacked as one-dimensional $N \times N_p$ vectors.

As (1) is ill-posed in general, a surrogate problem is solved instead, for example, problem (2).

2 $$\begin{aligned} \underset{x}{{\text {argmin}}} \; \left\{ \left\| P x - d \right\| _2^2 + \phi (x) \right\} \end{aligned}$$

where $\left\| \cdot \right\| _2^2$ is the squared Frobenius norm and $\phi (x)$ is a function bringing stability to the solution. A well-known example of such methods is the Total Variation minimization, which promotes images with sparse gradient.

In local tomography, problem (1) is even more ill-posed due to the incompleteness of the data *d*, as explained in the introductory part. In order for the solution to be acceptable, the exterior of the ROI has to be estimated. This can be done by extending the support of *x* to iteratively estimate the exterior by solving (3)

3 $$\begin{aligned} \underset{\tilde{x}}{{\text {argmin}}} \; \left\{ \left\| C \tilde{P} \tilde{x} - d \right\| _2^2 + \phi (\tilde{x}) \right\} \end{aligned}$$

where $\tilde{x}$ is an image with extended support $N_2 \times N_2 = N_2^2$, where $N_2 > N$, and $\tilde{P}$ is a wider projector adapted to this new geometry. To compute the data fidelity term (here the Euclidean distance between $\tilde{P} \tilde{x}$ and *d*), the size of the projected solution has to be consistent with the acquired data. Thus, the projection is cropped by the means of an operator *C* to recover the original local geometry. The cropping operator *C* maps an extended sinogram of support $N_2 \times N_p$ to a sinogram of support $N \times N_p$, by keeping only the *N* central columns. This models the truncation in the local tomography setup, where the detector is not large enough to image the entire object support $N_2$. In practice, the cropping operation is implemented inside the projector $\tilde{P}$ by simply restricting the projection to the detector limited field of view *N*. In the formulas, the cropping operator *C* is explicitly separated from the projector $\tilde{P}$ to highlight the local setup in the forward model.

Efficient implementations of the projection and backprojection operators enable to solve problem (3). The ASTRA toolbox [11], for example, has versatile geometry capabilities and built-in algorithms for solving (3) for $\phi (x) = 0$.

In this work, we consider the case where a subregion is known. This prior knowledge on the volume can be used to constrain the sets of solutions. A uniqueness theorem was stated in [6] along with a reconstruction algorithm based on differentiated backprojection and projection onto convex sets to invert the finite Hilbert transform. This algorithm, however, is difficult to implement, and no implementation is readily available for experiments.

We focus on a simpler approach based on formalism (3). In this formulation, the prior knowledge can be encoded in several ways. The first is to enforce the values of $\tilde{x}$ in the known region, for example, using an indicator function. The second is to add a term penalizing the distance between the values of $\tilde{x}$ in the known region and the actual values. We adopt the latter approach, which was proposed, for example, in [12].

Let $\Omega$ denote the domain where the values of the volume are known. It is a subset (possibly a union of subsets) of the image support $N^2$, and we denote $N_\Omega$ its cardinality, that is, the total number of known pixels. Let $x_{|\Omega }$ denote the values of *x* inside the known region. The prior knowledge is encoded by $\phi (x) = \lambda \left\| x_{|\Omega } - u_0 \right\| _2^2$, where $u_0$ denotes the known values inside $\Omega$ and $\lambda \ge 0$ is a parameter weighting the fidelity to the known zone. Both $x_{|\Omega }$ and $u_0$ have $N_\Omega$ components.

**Fig. 5:**
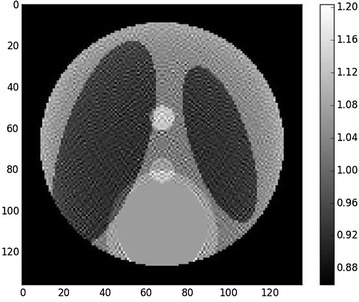
Result of iterative reconstruction with standard least squares minimization. Contrast was adapted with respect to the center of the image

**Fig. 6:**
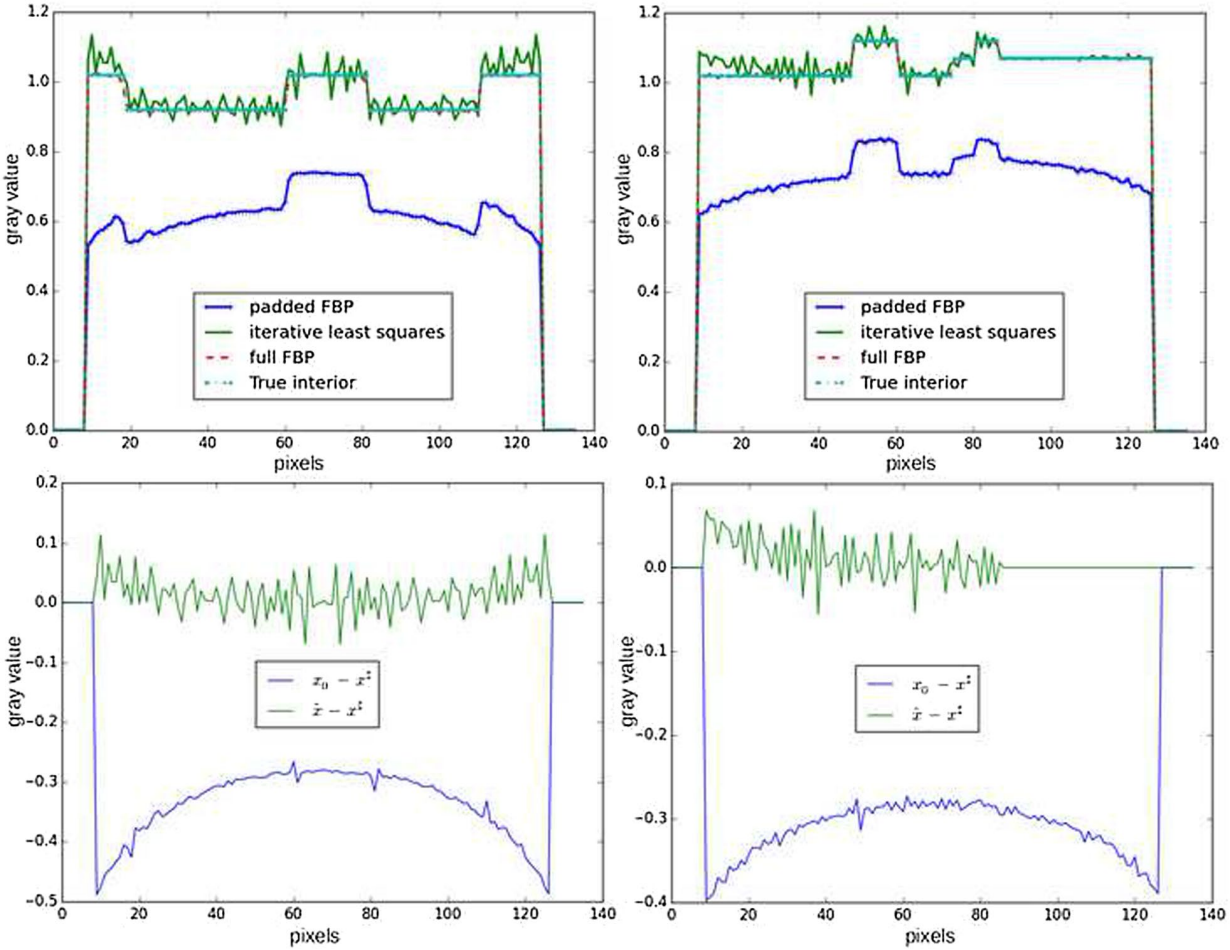
Line profiles of reconstructions of the Shepp–Logan phantom in a local tomography setup. *Top line* reconstruction profiles for padded FBP (*blue*) and iterative least squares (*green*). *Left* middle line of the reconstructed image. *Right* middle column of the reconstructed image. *Bottom line* difference profiles between the ground truth $x^\sharp$ and the reconstructions with padded FBP $x_0$ (*blue*) and iterative least squares $\hat{x}$ (*green*). *Left* middle line of the difference image, *right* middle column of the difference image. The iterative least squares reconstruction almost removes the cupping effect, but a high-frequency noise can be seen in the profiles

Figures 5 and 6 show the reconstruction result on the Shepp–Logan phantom with such choice of $\phi (x)$. The cupping effect is almost removed, but the reconstructed slice is also noisy, which is a known effect of least squares minimization on an ill-posed problem when running too many iterations [13]. On the other hand, many iterations are required to reduce the cupping effect.

**Fig. 7:**
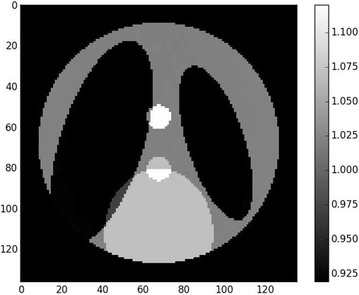
Result of iterative reconstruction with total variation regularization. Contrast was adapted with respect to the center of the image

**Fig. 8:**
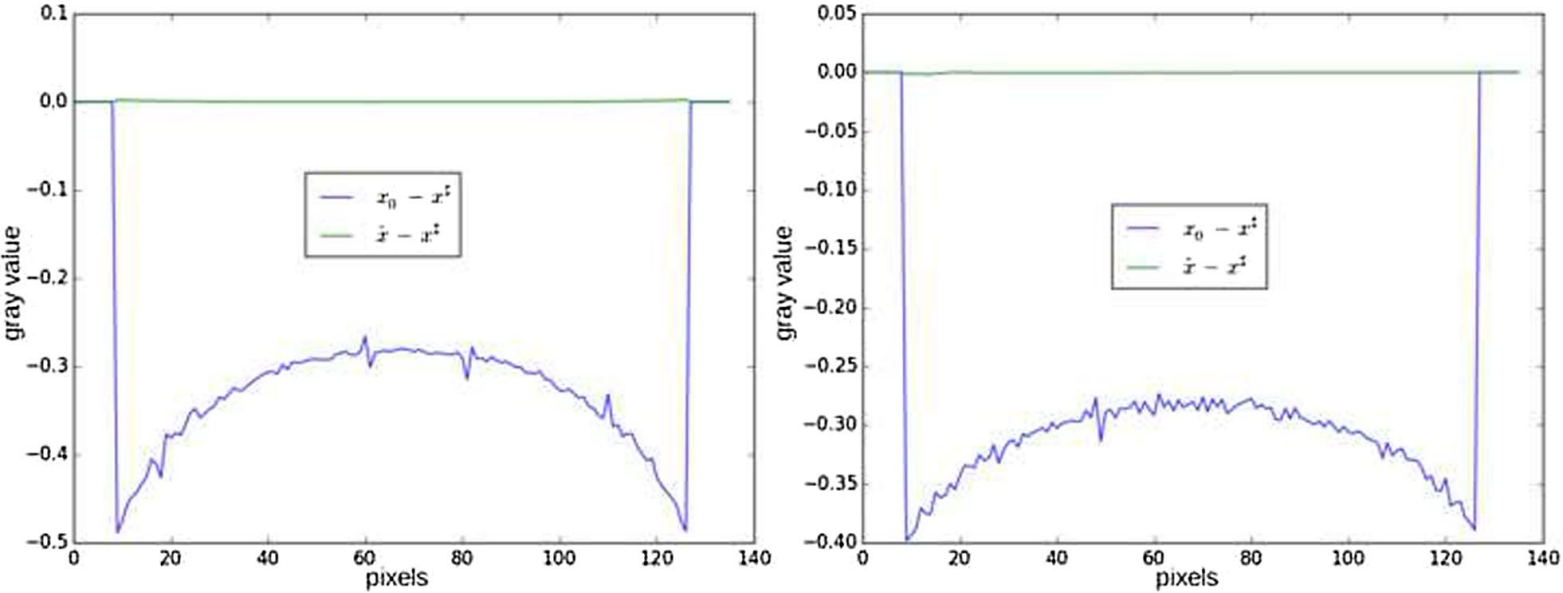
Line profiles of differences between reconstructions and ground truth. *Blue* difference between padded FBP $x_0$ and ground truth $x^\sharp$. *Green* difference between iterative total variation minimization $\hat{x}$ and ground truth $x^\sharp$. *Left* line profile, *right* column profile. Total Variation minimization removes both high- and low-frequency errors, exactly recovering the region of interest

A workaround on this problem is adding a regularization term to stabilize the solution. A popular regularization is Total Variation (TV), promoting piecewise-constant solutions. The function $\phi (x)$ in (3) can then be written $\phi (x) = \lambda \left\| x_{|\Omega } - u_0 \right\| _2^2 + \beta \left\| \nabla x \right\| _1$, where $\beta \ge 0$ weights the regularization. Figures 7 and 8 show the result of reconstruction with this method. The reconstruction is much more accurate and bears almost no difference with respect to the ground truth, which is an illustration of the uniqueness theorem stated in [6].

This approach, however, has two drawbacks. The first is using a prior which might not be accurate: in this example, Total Variation promotes piecewise-constant images and is thus not adapted for complex samples. The second drawback is on the computational side. Adding a non-differentiable prior involves to change the optimization algorithm for another probably less efficient in the sense that more iterations are required to reach convergence. In the examples, the preconditioned Chambolle–Pock algorithm described in [14] was used for the TV minimization. Approximatively, 3000 iterations are required to approximately get rid of the cupping effect (when approximatively 500 are required in the case of a complete scan), and more than 10,000 iterations are required to get the line profiles shown in Fig.  8. This approach is impracticable for modern datasets with increasing amount of data: on the one hand, projection and backprojection become costly operations, while on the other hand, even more iterations are required due to the higher number of variables.

The main contribution of this work is an efficient implementation of the method described in [9]. The method is based on the following observation: the padded FBP reconstruction yields acceptable reconstruction of features of the ROI [15], but can suffer from a low-frequency bias (cupping effect). On the other hand, iterative algorithms converge slowly due to the high indeterminacy of the problem, even with a known subregion. For these reasons, a refinement of the initial reconstruction is computed rather than the complete solution.

### Correction of the low-frequency bias

#### Estimating the reconstruction error

Let $x_0$ be a reconstruction of the region of interest with the padded FBP technique and $x^\sharp$ be the true values of the region of interest. Both are slices of support $N^2$ pixels. The reconstruction error, unknown in practice, is denoted $e = x^\sharp - x_0$. This error mainly consists in low-frequency artifacts (the cupping effect).

In this work, we implement a high-performance version of algorithm described in [9], aiming at improving an existing reconstruction $x_0$ by removing the cupping effect. This is done by solving the problem described by a new forward model (4)

4 $$\begin{aligned} \underset{x_e}{{\text {argmin}}} \; \left\{ \left\| C \tilde{P} (\tilde{x_0} + x_e) - d \right\| _2^2 + \phi (x_e) \right\} \end{aligned}$$

where $x_e$ is a correction term added to the initial reconstruction. Here again, $\tilde{x_0}$ denotes an extension of the support of $x_0$, $\tilde{P}$ is a projection operator adapted to this extended geometry, and *C* is a truncation operator. As the initial reconstruction is constant, problem (4) can be rewritten as

5 $$\begin{aligned} \underset{x_e}{{\text {argmin}}} \; \left\{ \left\| C \tilde{P} x_e - f \right\| _2^2 + \phi (x_e) \right\} \end{aligned}$$

where $f = d - C \tilde{P} \tilde{x_0}$. Problem (5) can be understood as fitting the (approximate) reconstruction error *f*. As the reconstruction error in the ROI is $e = x^\sharp - x_0$, we can write

6 $$\begin{aligned} \tilde{e}&= \tilde{x^\sharp } - \tilde{x_0} \nonumber \\ \tilde{P} \tilde{e}&= \tilde{P} \tilde{x^\sharp } - \tilde{P} \tilde{x_0} \nonumber \\ C \tilde{P} \tilde{e}&= d - P x_0 \end{aligned}$$

where $\tilde{x^\sharp }$ denotes the whole volume, so that $d = C \tilde{P} \tilde{x^\sharp }$ models the local tomography acquisition. If $x_0$ is extended to $\tilde{x_0}$ by inserting zeros, then $C \tilde{P} \tilde{x_0} = P x_0$ as there is no contribution from the external part. However, the quantity of interest is the reconstruction error (*e*) in the ROI, not in the whole volume ($\tilde{e}$). Since the projection of *e* is different from the cropped projection of $\tilde{e}$, the term $d - P x_0$ only approximates the projection of the reconstruction error in the ROI. This quantity is nevertheless used as an approximation of the projection of the reconstruction error in the ROI. Once the optimal correction term $\hat{x_e}$ is found, the resulting reconstruction is simply computed as $x = \tilde{C}(\tilde{x_0} + \hat{x_e})$ where $\tilde{C}$ is a cropping operator in the image domain, mapping images of support $N_2^2$ to images of support $N^2$.

#### Reducing the degrees of freedom

The principle of the implemented method is to refine an initial solution of the local tomography problem, knowing that middle and high-frequency features are usually well recovered. By focusing on the low frequencies, the complexity of problem (3) can be reduced by solving a simpler problem. Complexity reduction is achieved by expressing the reconstruction error in a coarse basis.

Gaussian function was chosen as a representation basis. The reconstruction error *e* is estimated by $\hat{e}$ as a convolution between a finite discrete Dirac comb and a two-dimensional Gaussian function $g_\sigma$ defined by Eq. (7)

7 $$\begin{aligned} g_\sigma (u, v) = \frac{1}{\sigma \sqrt{2\pi }} \exp \left( -\frac{u^2 + v^2}{2\sigma ^2} \right) \end{aligned}$$

where $u, \, v$ denote discrete indexes in the image, and $\sigma > 0$ is the standard deviation characterizing the Gaussian function. The estimate of the reconstruction error at location $(u_0, v_0)$, $\hat{e}(u_0, v_0)$, is then given by Eq. (8)

8 $$\begin{aligned} \hat{e}(u_0, v_0) = \sum _{u, v} c_{u, v} g_\sigma (u_0 - u \cdot s, \, v_0 - v \cdot s) \end{aligned}$$

where $c_{u, v}$ are coefficients multiplying the Gaussian functions $g_\sigma$, and *s* is the spacing (in pixels) between points of the Dirac comb. The summation in (8) actually occurs on a finite support. In our implementation, the Gaussian function is truncated at $3\sigma$ at each side, so the sum takes place on a $\lfloor 6\sigma +1\rfloor \times \lfloor 6\sigma +1\rfloor$ pixels square.

Estimation (8) is done such that projection of $\hat{e}$ has minimal Euclidean distance with $d - P x_0$. Let *G* denote the operator mapping the coefficients $c_{i,j}$ to the image $\hat{e}$ through convolution formula (8). The coefficients vector *c* is estimated by solving Problem (9)

9 $$\begin{aligned} \underset{c}{{\text {argmin}}} \; \left\{ \left\| C \tilde{P} G c - f \right\| _2^2 + \phi (c) \right\} \end{aligned}$$

where $f = d - P x_0$ and $\phi (c)$ is a constraint function on the coefficients which is detailed later.

Thus, Problem (9) is solved instead of Problem (3). In Problem (9), the unknowns are the coefficients *c* of the coarse basis. As there are much less coefficients *c* in the coarse representation than pixels in the extended image support $N_2^2$, the degrees of freedom is accordingly reduced.

Solving (9) requires the computation of the operators *C*, $\tilde{P}$, *G*, and possibly their adjoints. The implementation of the crop operator *C* is straightforward, as it consists in truncating the sinogram to the size of the acquired data. In practice, it consists in modifying the projector $\tilde{P}$ so that the projections are limited to the reduced detector field of view $N_2$. The operator *G* can be described as follows. Coefficients $c_{u,v}$ are placed every $s > 0$ pixel on an image of the size of the extended reconstruction $\tilde{x_0}$. This image (a two-dimensional Dirac comb in the continuum case) is then convolved by the kernel $g_\sigma$. Lastly, an efficient implementation of the projection and backprojection operators is needed to solve (9). This is discussed in the implementation section.

#### Adding the known zone constraint

We now describe how the known zone constraint is implemented in formalism (9). In work [9], the knowledge available as known zone values in the slice is translated in the coarse representation basis: a subset of Gaussian coefficients is fitted to values in the known zone $\Omega$; these coefficients are then used as a constraint for the reconstruction.

In this work, we rather add the constraint directly in the pixel zone. The final optimization problem is

10 $$\begin{aligned} \underset{c}{{\text {argmin}}} \; \left\{ \left\| C \tilde{P} G c - f \right\| _2^2 + \beta \left\| G c_{|\Omega } - u_0 \right\| _2^2 \right\} . \end{aligned}$$

## High-performance implementation

After having reduced the number of degrees of freedom for problem (4), we describe an efficient implementation of the involved operators based on look-up tables (LUTs).

### Projection a of Gaussian tiling

The choice of a Gaussian basis for a coarse representation of the correction term is based on a characteristic of the Gaussian kernel: it is both rotationally invariant and separable [16]. These two properties provide a computational advantage: the order of projection and convolution can somehow be exchanged.

**Fig. 9:**
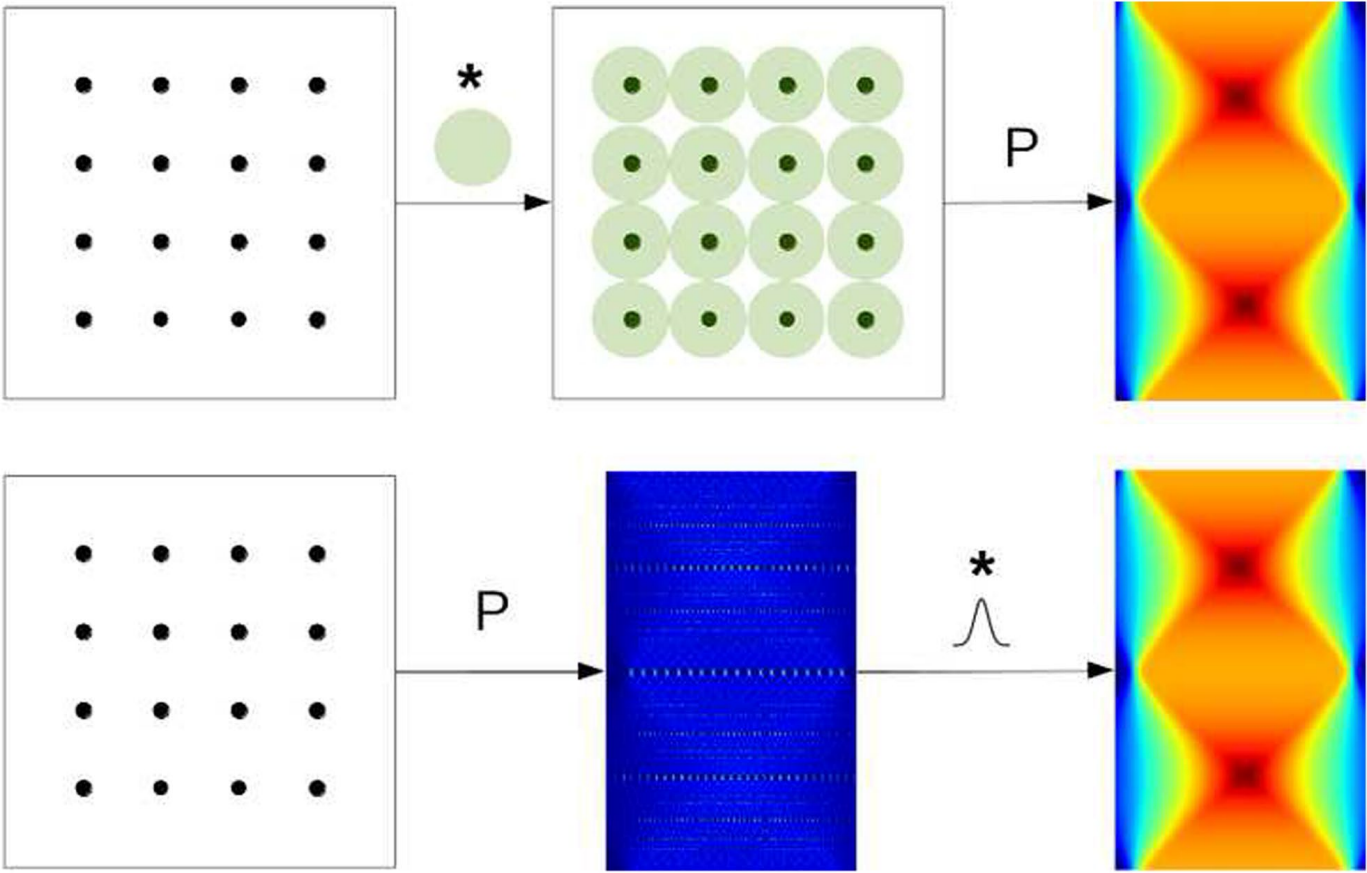
Illustration of the alternative way of computing the projection of a tiling of Gaussian functions. In the first approach (*top line*), coefficients are evenly placed on the image support (*left*). This image is then convolved by the 2D Gaussian kernel (*green circles*), which gives an intermediate image (*center*). This image is projected to obtain a sinogram (*right*). In the second approach (*bottom line*), isolated coefficients (*left*) are projected. Each line of the resulting sinogram (*middle*) is convolved by a 1D Gaussian kernel, to obtain the sinogram (*right*)

More precisely, given an image *y* consisting of the Gaussian coefficients evenly placed with a spacing *s*, the standard way to compute $\tilde{P} G y$ is first performing the convolution *Gy* defined by (8) and then projecting with $\tilde{P}$. An equivalent computation, however, can be done by first projecting the image of isolated points *y*, and then convolving each line of the resulting sinogram by a one-dimensional Gaussian function. This is illustrated in Fig. 9.

This latter approach has two advantages. First, the two-dimensional convolution (or two series of one-dimensional convolution in this separable case) is replaced by a series of one-dimensional convolutions. Secondly, the projection here only consists in projecting isolated points. This operation can be optimized by designing a *point-projector* based on look-up tables.

### LUT-based point-projector

As previously discussed, the operators involved in forward model (10) are a cropping operator, a one-dimensional convolution, and a projector. The convolution can be efficiently implemented, either in the Fourier space or in the direct space when one of the functions has a small support. Therefore, a fast projector is essential for solving (10) in an iterative fashion. In our case, the object to project has a very special structure, as it consists in points spaced by several pixels. Thus, standard projectors of tomography softwares can be replaced by a more efficient implementation, hereby called *point- projector*, based on look-up tables.

In the remainder, the following notations are used: The support of the original image is $N^2$. The number of projections is $N_p$, so the acquired sinogram has size $N \times N_p$. The size of the extended image is $N_2 ^2$ where $N_2 \ge N$. The number of Gaussian functions used to tile the support is $N_g$. The spacing between Gaussian blobs on the image is *s* ; thus we have $N_g \simeq \left( \frac{N_2}{s} \right) ^2$ in a first approximation. We also use the following indexes convention : Gaussian coefficients are numbered with $i \in [0, N_g]$, and sinogram indexes are numbered with $k \in [0, N_s]$ where $N_s = N_2 \times N_p$ is the size of the (extended) sinogram.

**Fig. 10:**
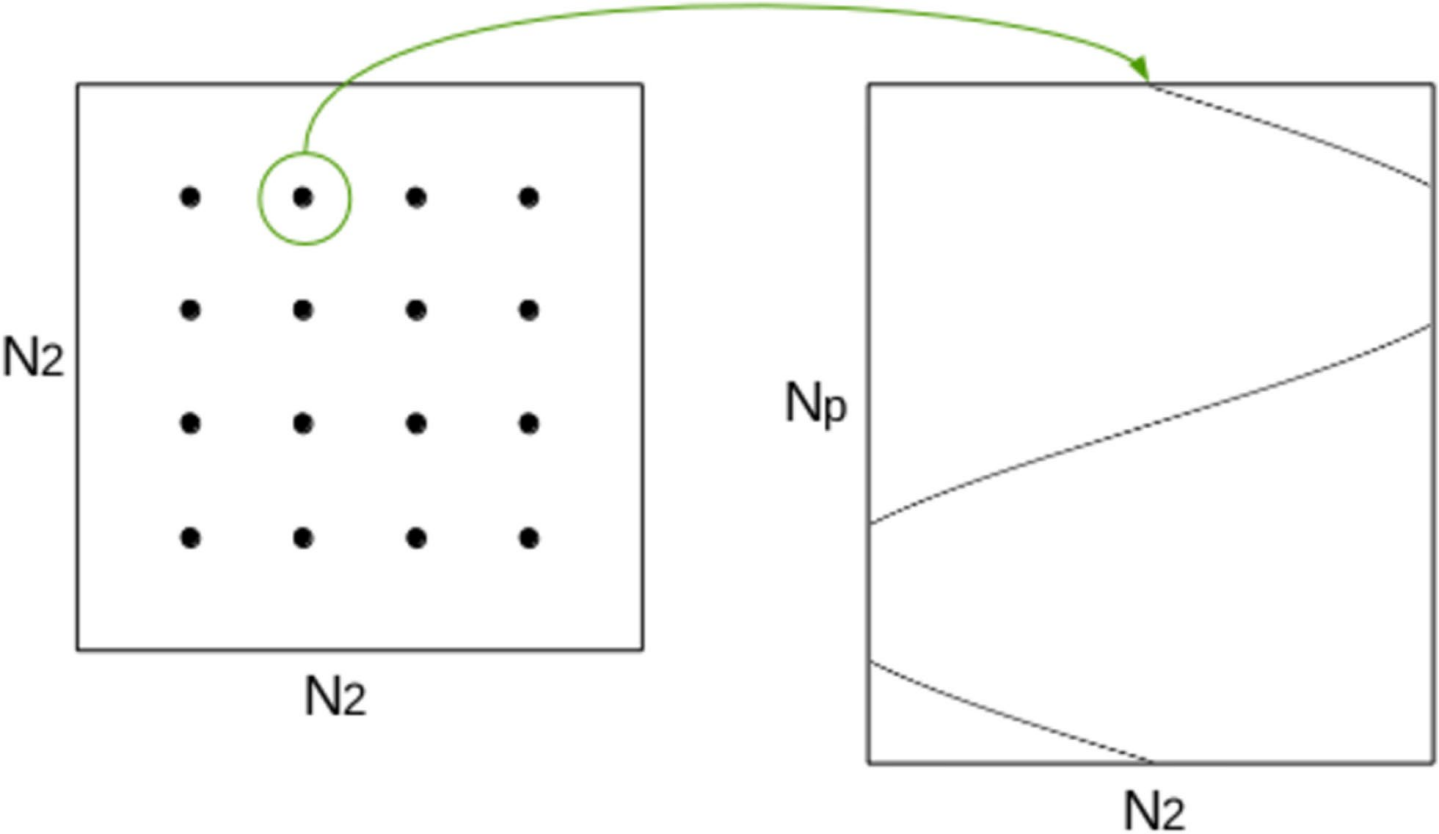
Principle of the point-projector. Gaussian basis coefficients are placed on the image of $N_2 ^2$ support (*left*), with even spacing. Each isolated point is projected on at most $N_p$ positions in the sinogram (*right*)

**Fig. 11:**
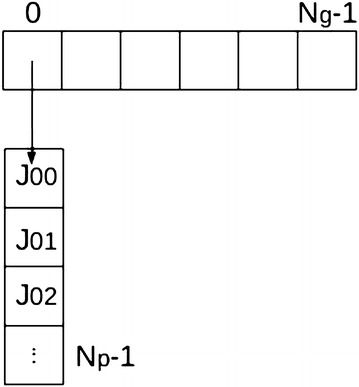
Illustration of the look-up table *J*. The Gaussian coefficients placed on the image are stored in a vector of size $N_g$ (*top*). Each coefficient point (indexed in $[0, N_g]$) is projected on at most $N_p$ positions in the sinogram. For each $i \in [0, N_g]$, the structure $J[i] = \left\{ J_{i,0}, \, J_{i,1}, \, \ldots \right\}$ (*bottom*) contains the list of the sinogram positions hit by projection of *i*. For example, the Gaussian coefficient number 0 is projected on sinogram positions $J_{0, 0}, \, J_{0, 1} \, \ldots$

Each Gaussian coefficient number $i \in [0, N_g[$ is projected on (at most) $N_p$ positions in the sinogram. Therefore, a look-up table *J* is built so that for each *i*, *J*[*i*] is the “list” of locations in sinogram hit by this point after projection. The LUT *J* is an array of size $N_g \times N_p$. Each entry $J_{i,j}$ corresponds to a position, in the sinogram, that is hit by a projected point $i \in [0, N_g]$. For example, entry $J_{0,2}$ is an index in the sinogram that is hit by point 0 ; and entry $J_{5,j}$ are an indexes hit by point 5 for all *j*. This is illustrated in Figs. 10, 11.

**Fig. 12:**
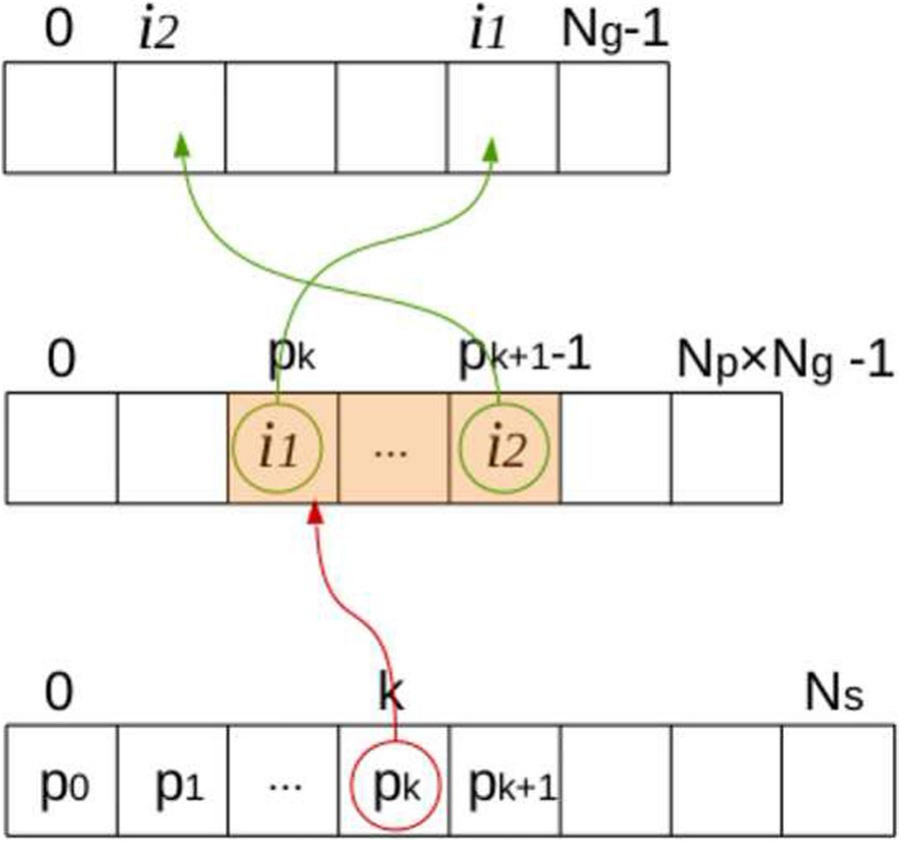
Illustration of the LUT-based point-projector. To determine which points are projected on position $k \in [0, N_s]$ of the sinogram, the matrix $\mathrm {Pos}$ (*bottom*) is accessed at index *k*, and contains the value $\mathrm {Pos}[k] = p_k$. This value $p_k$ is a position in LUT *J* (*middle*), so that $J[p_k] = i_1$ is the index of one coefficient being projected at index *k* in sinogram. The process is repeated for $p_k +1$, until $p_{k+1}-1$. The corresponding range in *J* (*shaded orange*) indicates coefficient indexes that all are projected on sinogram index *k*.


When computing the sinogram, however, the look-up table *J* is best accessed “backward”: for a given position $k \in [0, N_s[$ in the sinogram, we have to determine which points are hitting it through projection. To this end, two look-up tables *J* and $\mathrm {Pos}$ are built. For $k \in [0, N_s]$, $\mathrm {Pos}[k]$ indicates a position in LUT *J*, and $J[p_k]$ is a coefficient number $i \in [0, N_g]$ being projected at position *k*. Therefore, the LUT *J* does not contain sinogram indexes anymore, but rather coefficient indexes. This is illustrated in Fig. 12. The LUT *J* is re-ordered such that the interval $[p_k, p_{k+1}-1]$ gives access to an indexes range in *J* ; this index range is the set of all coefficients indexes being projected on sinogram index *k*.

The point-projector is described by Algorithm 1. The matrix *W*, indexed in the same way as *J*, contains the weights of the projections: depending on the position of a point in the image and the projection angle, its projection does not exactly fall into a sinogram pixel. The matrix *W* thus encodes the geometric contribution of the projection of the points.

This projection scheme basically consists in storing the explicit projection matrix $\tilde{P}$ with a *Compressed Sparse Row* (CSR) format [17], where LUT *J* corresponds to “col_ind,” LUT $\mathrm {Pos}$ corresponds to “row_ptr,” and matrix *W* contains the values. Storing the entire “linear-algebra” projection matrix without compression would entail to store $(N_2^2)\times (N_2 \cdot N_p)$ elements, which is impracticable (for example, more than one terabyte is required for a $1024^2$ slice). However, as each slice point is projected on at most $N_p$ sinogram positions, this matrix actually has at most $N_2^2 \times N_p$ non-zero elements. Additionally, as the slice is reduced on a coarse basis, there are $\left( \frac{N_2}{s} \right) ^2 \times N_p$ non-zero values to store in this case. The format described above is used to store these elements. Algorithm 1 is thus no more than a matrix-vector multiplication with a sparse matrix in CSR format.

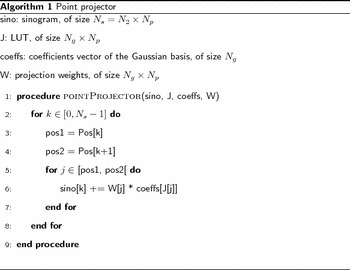


This approach for computing the point-projector is friendly in a memory-write point of view: after accumulating the contributions of all coefficients projected on position *k*, the sinogram at index *k*, $\mathrm {sino}[k]$, is updated accordingly. This is especially important for GPU implementation, as consecutive threads access contiguous memory locations, which is a coalesced access pattern. On GPUs, each memory transaction actually entails accessing *L* bytes, so coalesced access to 32 bits scalars results in a read or write of *L* / 4 addresses in a single transaction (for example, $L = 128$ for modern NVidia GPUs).

### Implementation of the adjoint operators

As a gradient-based optimization algorithm is used for solving (10), the adjoint of operator $C \tilde{P} G$ has to be computed. This operator $G^T \tilde{P}^T C^T$ consists in extending the sinogram with zeros, point-backprojecting and retrieving the Gaussian components from the backprojected image. As mentioned above, the operator *G* can be described as $G = H_\sigma U$ where *U* is an upsampling operator (here with a factor *s*), and $H_\sigma$ is the convolution with 2D Gaussian kernel (7). Thus, $G^T = H_\sigma ^T U^T$ which is a downsampling followed by a convolution with kernel (7). The actual computation is then $G^T \tilde{P}^T C^T = H_\sigma ^T U^T \tilde{P}^T C^T = U^T \tilde{P}^T H_\sigma ^1 C^T$ where $H_\sigma ^1$ is a one-dimensional convolution on the sinogram rows.

As previously, these operations can be merged. As $G^T \tilde{P}^T C^T$ returns a Gaussian coefficients vector from a sinogram, only the coefficients are of interest here. Therefore, the point-backprojector $\tilde{P}^T$ is merged with the downsampling $U^T$ as previously. For a given coefficient, we have to find which sinogram entries backproject on the coefficient position. This approach avoids to compute useless $N_g \times (s-1)^2$ backprojections points on the image, as it is downsampled afterward.

**Fig. 13:**
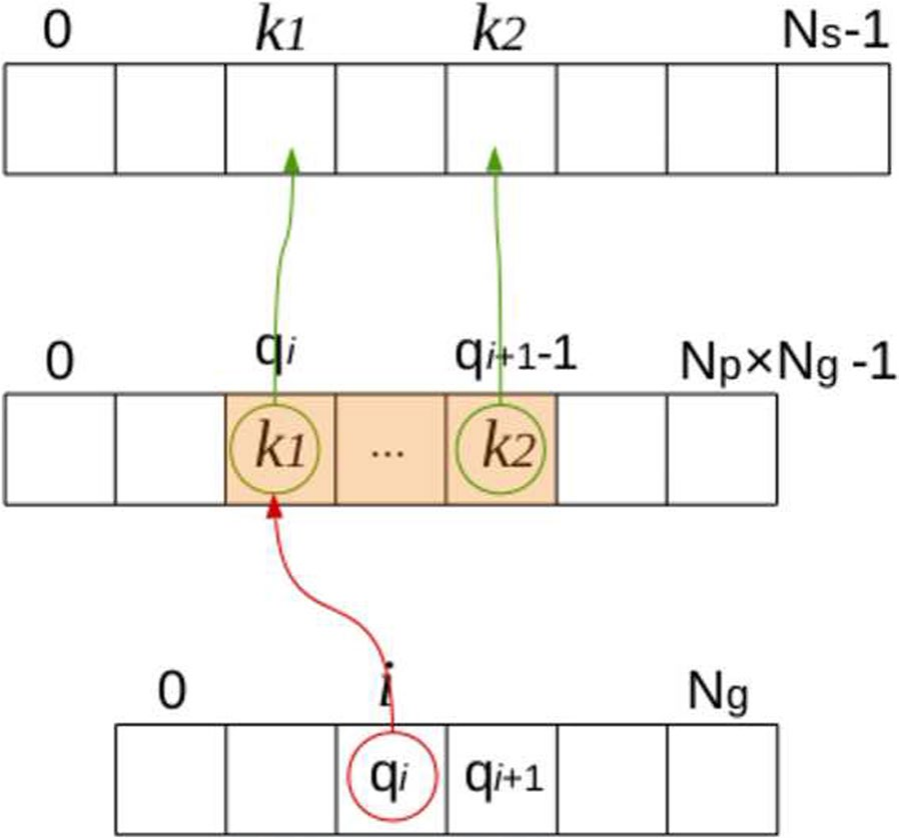
Illustration of the LUT-based point-backprojector. To determine which sinogram points are backprojected on coefficient $i \in [0, N_g]$, the matrix $\mathrm {Pos2}$ (*bottom*), is accessed at index *i*, and contains the value $\mathrm {Pos2}[i] = q_i$. This value $q_i$ is a position in LUT $J_2$ (*middle*), so that $J_2[q_i] = k_1$ is the index of one sinogram entry being backprojected at index *i* of the coefficients vector. The process is repeated for $q_i +1$ until $q_{i+1}-1$. The corresponding range in $J_2$ (*shaded orange*) indicates sinogram indexes that are all backprojected on coefficient index *i*

The point-backprojector is implemented, as previously, with a LUT $J_2$ of size $N_g \times N_p$ and a LUT $\mathrm {Pos2}$ of size $N_g +1$. The matrix $\mathrm {J_2}$ is re-ordered so that for all $i \in [0, N_g[$, the interval $[\mathrm {Pos2}[i], \, \mathrm {Pos2}[i+1]-1]$ corresponds to an index range in LUT $J_2$. This is illustrated in Fig. 13.

The point-backprojector is given by Algorithm 2. Again, the backprojection from a sinogram to a Gaussian coefficients vector corresponds to a matrix-vector multiplication with a matrix in CSR format. The matrix $W_2$, containing the geometric weights of the backprojector, can be viewed as the Column Sparse Storage (CSC) version of the matrix *W*.
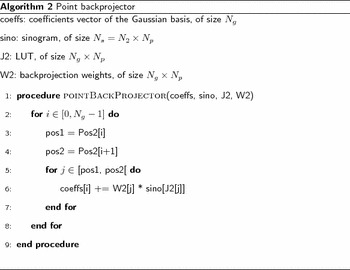


### Parallel implementation

In modern experiments carried on X-ray light sources, the data volumes, produced by new generations of detectors, always overwhelm the computing power. Simply waiting for more powerful machines is of little hope, as advances in detectors overrun the Moore’s law. Instead, an algorithmic work has to be accomplished to exploit parallelism of modern architectures. In the last decade, the advent of general-purpose GPU (GPGPU) computing was advantageously used, especially in tomography.

The proposed method has been implemented in the PyHST2 software [18] used at ESRF for tomographic reconstruction, with the CUDA language targeting Nvidia GPUs. The point-projector and point-backprojector, which are the most time-consuming operators, are implemented as efficient CUDA kernels. As for Algorithms 1 and 2, the CUDA point-projector and point-backprojector are implemented as matrix-vector multiplication with a matrix in CSR format.

**Fig. 14:**
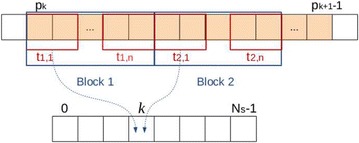
Illustration of the GPU LUT-based point-projector. The memory range $[p_k, p_{k+1}-1]$ in LUT *J* (*top*, *shaded orange*) contains all the indexes needed to be accessed in the coefficients vector to compute $\mathrm {sino}[k]$. In this illustration, each thread reads $m = 2$ values in the LUT (*red rectangles*). The threads are grouped in blocks of *n* threads (*blue rectangles*). In the block 1, threads $t_{1, 1}, \ldots , t_{1, n}$ update a temporary shared array with their contribution. The same is done in block 2, where another temporary shared array is used. Then, one thread per block accumulates the results of the shared array and adds the results to $\mathrm {sino}[k]$. The addition has to be atomic, as threads from several groups might access $\mathrm {sino}[k]$ at the same time.

We describe here the implementation of the point-projector, i.e., the computation of the sinogram values $\mathrm {sino}[k]$ for $k \in [0, N_s]$. The point-backprojector follows the same principle. To compute the sinogram value $\mathrm {sino}[k]$, the LUT *J* has to be accessed from $p_k$ to $p_{k+1}-1$ as illustrated in Fig. 13. This memory range is accessed in parallel by threads of the many-cores GPU with the following principle. Each thread reads $m \ge 1$ values in the LUT. With these values *J*[*j*], where $j = p_k, p_k +1, \ldots$, the coefficients vector is accessed at $\mathrm {coeffs}[J[j]]$. The threads are grouped in blocks, and each thread updates a temporary array in shared memory with the contributions read in $\mathrm {coeffs}[J[j]]$. Then, in each block, the shared array is accumulated by one thread. The result is added to $\mathrm {sino}[k]$. This is illustrated in Fig. 14.

The parallelization is done on the read of matrix *J*, as it is the biggest data structure of the method. As it has been re-arranged so that the interval $[\mathrm {pos}[k], \mathrm {pos}[k+1]-1]$ is a contiguous memory range in *J*, the described implementation has an efficient memory access pattern.

### Multi-resolution Gaussian basis

The correction term $x_e$ in model (4) is a tiling of Gaussian functions : $x_e = G c$, where *c* is the vector of coefficients in the Gaussian basis, and *G* is the operator previously described. In a first approach, all the Gaussian functions (7) have the same variance $\sigma ^2$, so that operator *G* is linear and problem (10) is convex. The coefficients are placed on a support of size $N_2^2$ before being (theoretically) convolved with a 2D Gaussian kernel. The spacing between points is *s*, so that the number of required coefficients is approximately $N_g \simeq \left( \frac{N_2}{s} \right) ^2$.

Another approach is using different variances depending on the position in the image. As only the support $N \le N_2$ of the original reconstruction $x_0$ has to be corrected, Gaussians with a larger support (larger $\sigma$) can be used on the exterior of the ROI, further reducing the number of unknowns. By using small Gaussians (small $\sigma$) inside the ROI, local features can be estimated in the correction term $x_e$, while large Gaussians are used to roughly estimate the contribution of the exterior of the ROI. The new operator *G* can be written

11 $$\begin{aligned} G = \sum _j H_{\sigma _j} U_j \end{aligned}$$

where $\sigma _1, \sigma _2, \ldots$ is a series of standard deviations for the Gaussians, and $U_j$ are upsampling operators with different factors. This representation is similar to a multi-resolution scheme also used in [19]. This multi-resolution basis allows to further reduce the number of variables in vector *c*: in this case, $N_g < \left( \frac{N_2}{s} \right) ^2$. In our implementation, the standard deviations are progressively doubled until reaching the diameter of the ROI and then remain constant outside the ROI.

Implementation of (11) is straightforward. The coefficients in vector *c* are classified according to the distance to the center, forming subsets of coefficients $c^1, c^2, \ldots$. Each subset is point-projected and line-convolved with the corresponding $\sigma _1, \sigma _2, \ldots$. The resulting sinograms are summed to obtain the projection of *Gc*.

This representation of correction features as Gaussian blobs is actually not a basis in the mathematical sense: some images cannot be represented by a linear combination of Gaussians. However, this representation is very close to a basis for $\sigma \simeq s$ [20]. In our case, we choose $s = 0.65 \times \sigma$, meaning that there is a significant overlap between the Gaussians. The discrete Gaussian kernel is truncated at $3\sigma$, so its length is $\lceil 6\sigma +1 \rceil$ samples.

### Optimization algorithm

Efficient optimization algorithms can be used to solve the quadratic problem (10). We use the conjugate gradient (CG) algorithm, requiring the computation of the adjoint of the involved operators previously described. CG also entails matrix-vector multiplications, which are efficiently implemented with the CSR representation of point-projector and backprojector.

In the GPU implementation, all the involved arrays are single precision (float 32 bits) as most GPUs are relatively not efficient with 64 bits operations. However, the conjugate gradient algorithm involves scalar products. These operations are implemented by dedicated kernels returning double precision values, as error accumulation is noticeable when accumulating on large arrays in single precision.

## Results and discussion

**Fig. 15:**
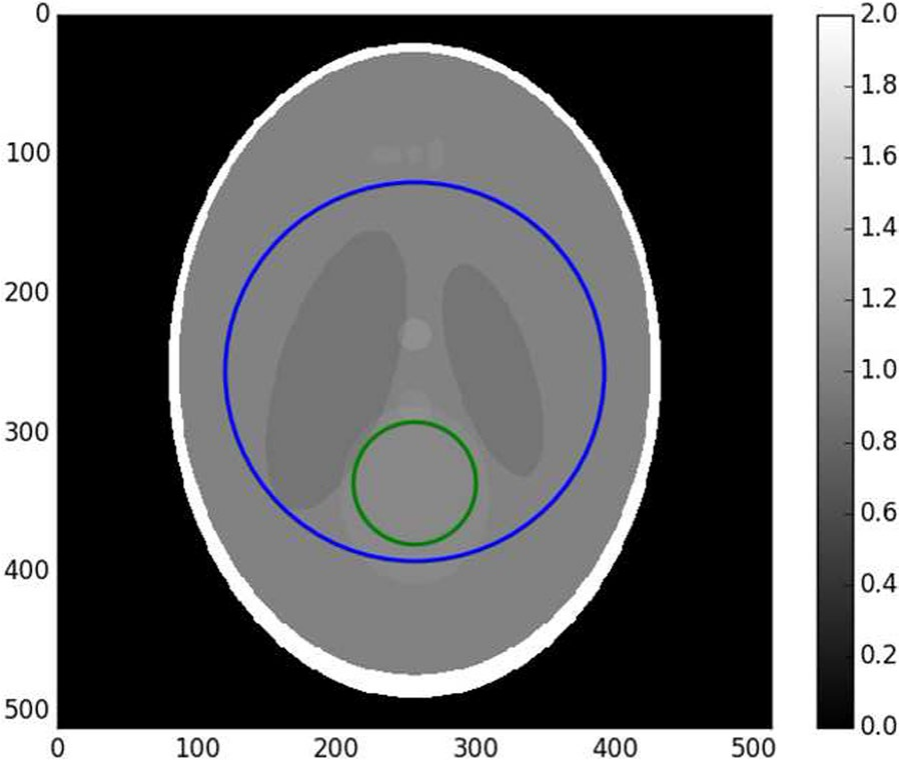
Example of local tomography setup on the $512^2$ *Shepp–Logan* phantom. The outer circle (*blue*) is the region of interest. The inner circle (*green*) is the known subregion.

In this section, we compare the proposed method with the basic exact method described in the second section, in term of speed and correction capabilities. The speed benchmarks are done with the low- resolution Shepp–Logan phantom, as the cupping effect is prominent in this image. The setup is illustrated in Fig. 15. The size varies in the benchmarks, and the radii of ROI and known zone also vary accordingly. The known zone has been chosen as a uniform zone. In experimental datasets, the known region can be for example regions where the sample contains air, for which the attenuation coefficient is known to be zero.

**Fig. 16:**
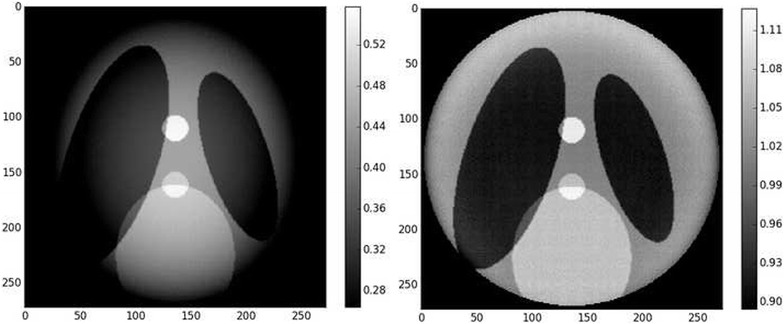
ROI reconstruction results. *Left* padded FBP, *right* proposed. For both images, the contrast was adapted with respect to the center.

**Fig. 17:**
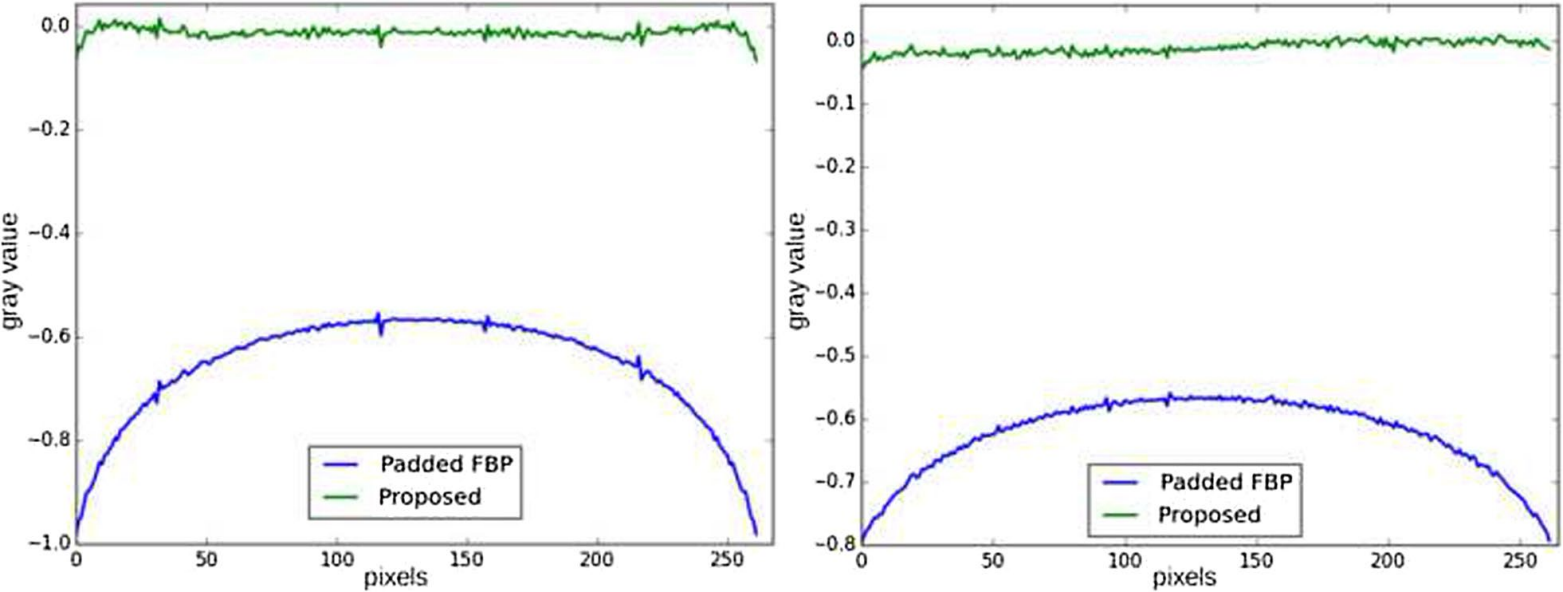
Line profiles of reconstructions with the proposed method and the padded FBP. The proposed method was executed with $N_g = 1345$ (*left*) and $N_g = 729$ (*right*), corresponding to a relatively coarser basis.

Figure 16 shows the reconstruction results for the setup of Fig. 15. As it can be seen, the cupping effect is mostly removed with respect to the padded FBP technique. Importantly, the proposed method does not create additional artifacts when correcting the cupping effect. Figure 17 shows line profiles of these reconstructions. The known zone constraint provides a reconstruction with an almost zero mean bias.

In the following benchmark, the following notations are used. *N* is the horizontal size of the initial reconstruction, i.e., the diameter of the acquired ROI, which means that the acquired sinogram has a size $N \times N_p$. $N_0$ is the horizontal size of the whole object support, unknown in practice (for example, $N_0 = 512$ in the case of the $512^2$ Shepp–Logan phantom). $N_2$ is the horizontal size of the extended reconstruction ($N_2 > N$), which should approximate $N_0$. Lastly, $N_g$ is the number of Gaussian functions used for the proposed method.

All the tests were performed on a machine with a Intel Xeon CPU E5-2643 12 cores 3.40 GHz and a Nvidia Geforce GTX Titan X GPU. As the LUT can be used for all the slices of a volume, the computation of the LUT is not taken into account. The optimization algorithms used are the preconditioned Chambolle–Pock method [14] for pixel domain exact method and Conjugate Gradient for the proposed method.

We report both the number of iterations needed to converge to the objective function minimum and the total execution time. This gives information on both the efficiency on the optimization algorithm to converge for the given problem and on the complexity of each iteration, as the time for one iteration is roughly the total execution time divided by the number of iterations.

**Table 1:** Execution time for various local tomography setups

$N_0$	*N*	$N_2$	$N_g$	Its	Time (s)	PSNR	Its	Time (s)	PSNR
512	272	572	1345	200	10.2/2.31	35.5	4000	123	36.79
512	272	572	729	200	5.86/2.01	34.93	3000	106	35.94
1024	544	1144	1345	300	36.71/5.14	28.03	4000	523	31.56
1024	544	1144	2081	300	60.7/11.4	30.25	8000	1094	37.85
2048	1088	2288	1345	500	235/33.5	27.73	4000	3570	15.13
2048	1088	2288	805	500	129/19.2	24.75	7000	6237	20.71
4096	2176	4576	2081	500	1028/109	24.11	4000	14920 (E)	N.A.
4096	2176	4576	1037	500	870/97.6	22.74	7000	26110 (E)	N.A.

The columns ($N_0$, *N*, $N_2$, $N_g$) describe the problem setup. The next three columns indicate the results for the proposed method. The next three columns indicate the results for the exact method compared against. The first “Time (s)” column contains the execution time for CPU and GPU, in the form. The second ``Time (s)" column contains the execution time for the other method. Values followed by (E) were extrapolated from previous running times.

Table 1 summarizes the results of the two methods for various setups. For each original phantom size, the two methods are tested with two sets of different parameters. For $512^2$, $1024^2$, $2048^2$, and $4096^2$ original phantom shapes, the number of projections are, respectively, 800, 1500, 2500, and 4000.

The prototype of [9] was run with the parameters of Table 1. It yields the following execution times: 11.3 s for a $512^2$ image, 83.1 s for a $1024^2$ image, 842 s for a $2048^2$ image, and 3630 s for a $4096^2$ image. Although it is still better than the “pixel domain approach,” it suffers from very long execution times for large images.

In the example of $512^2$ phantom size, the proposed method is executed with an acquired sinogram of width 272 pixels. The slice is extended to 572 pixels, and the Gaussian basis is configured to have 1345 functions in total. 200 iterations yield the reconstruction of Fig. 16 in 10.2 s (without taking the LUT computation time). On the other hand, the standard pixel domain method is executed with 4000 iterations and yields a reconstruction similar to Fig. 7, although of slightly lesser quality, in 123 s. The test is then run for a smaller number of Gaussians: the execution time is reduced, but the quality is slightly degraded. This is due to the fact that the number of Gaussians is determined by the spacing *s*, which itself is linked to the standard deviation $\sigma$. Decreasing the number of unknowns ($N_g$) speeds up the computations and also increases the width of the Gaussians, so the reconstruction error might not be appropriately fitted.

**Fig. 18:**
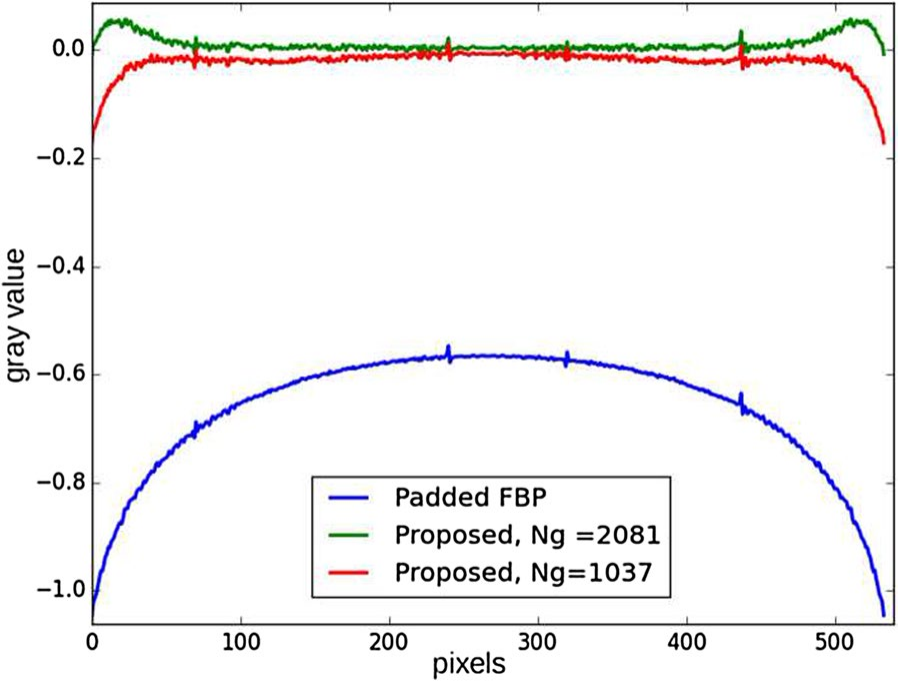
Line profile of reconstruction of a $1024^2$ phantom with $544^2$ pixels ROI, with different numbers of Gaussian functions

The parameters of the proposed method are essentially the size of $N_2$ of the extended slice and the initial value for $\sigma$. As explained in the multi-resolution subsection, the standard deviations are then progressively doubled until reaching the ROI radius and then kept to a maximal value outside the ROI. Given a size $N_2$, small initial $\sigma$ leads to larger computation times as there are more functions in the basis, so the LUTs are bigger. Larger initial $\sigma$ decreases the computation time but might yield coarser results. Figure 18 shows an example of the influence of the number of Gaussians $N_g$ on the result in the case of a $1024^2$ original phantom. As seeing the profile, the cupping removal is slightly better when $N_g$ is bigger (smaller Gaussians), and the error profile is overall closer to zero.

The exact method with pixel domain variables starts to be impracticable from $2048^2$ pixels slices , as thousands of iterations are required to yield an acceptable image quality, leading to hours of processing per slice. The execution times for $4096^2$ slices were extrapolated from the measured time on 100 iterations: 373 s; therefore, the PSNR are not available in these cases. This method is actually implemented in Python with the ASTRA Toolbox, meaning that only the projection and backprojection are performed on GPU, so the implementation suffers from memory transfers between CPU and GPU. If fully implemented on GPU, one could expect a 5–10 speed-up for this method; nevertheless, the proposed method would still be ahead.

For both methods, the PSNR is progressively decreasing as the size of the slice increases, yet the reconstructions are satisfying. We believe this is a consequence of the cupping being not entirely corrected on the slice borders, which brings more and more contribution as the number of pixels increase.

The current GPU implementation provides acceptable speed-up with respect to the CPU implementation, but there is certainly room for improvement as many parts in the CPU implementation are single-threaded. The computation of the LUT takes several minutes for $4096^2$ slices, but the same LUT is re-used for all the slices of a volume.

## Conclusion

We proposed a high-performance implementation of a local tomography method aiming at removing the cupping effect. The method consists in iteratively correcting an already reconstructed slice and to reduce the reconstruction error in a Gaussian blobs basis. This implementation is based on a careful analysis of the optimization process, showing that the involved operators can be designed especially for this problem.

Results validate the implementation on simulated data, showing that the known zone constraint effectively enforces an almost zero bias. Benchmarks show that $4096^2$ slices can be processed in tens of seconds, making it able to cope with modern data volumes.
